# Perceived parental involvement influences students’ academic buoyancy and adaptability: the mediating roles of goal orientations

**DOI:** 10.3389/fpsyg.2023.1248602

**Published:** 2023-08-03

**Authors:** Mudan Chen, Ida Ah Chee Mok

**Affiliations:** Faculty of Education, The University of Hong Kong, Pokfulam, Hong Kong SAR, China

**Keywords:** academic buoyancy, adaptability, goal orientations, parental involvement, mathematics learning

## Abstract

Academic buoyancy and adaptability (i.e., student capacities to deal with difficulties and challenges in daily school lives and to make appropriate cognitive, behavioral as well as affective adjustments in interacting with new, uncertain, and/or changing situations, circumstances, and conditions) can help learners regulate and protect themselves in the failure-prone learning environment. This study examined how students’ perceptions of parental academic involvement and their goal orientations were related to their academic buoyancy and adaptability in mathematics learning. We recruited a sample of 1,164 Chinese junior high school students. Using structural equation modeling, the results indicated that after controlling for family socioeconomic status and gender, perceived parental involvement was positively related to the students’ academic buoyancy and adaptability. Furthermore, parental involvement was significantly associated with students’ mastery and performance-approach goal orientations, which further partially mediated the relationship between parental involvement and academic buoyancy and adaptability. However, the mediating role of a performance-avoidance goal orientation in this relationship was not significant. Findings highlight the important roles that parenting practices and individual achievement motivation play in the development of academic buoyancy and adaptability in the Chinese context. Future research directions and implications are discussed.

## Introduction

A vast majority of students may encounter difficulties, challenges, changes, and uncertainties throughout their daily school lives, which calls for day-to-day coping abilities. In the subject of mathematics, the complexity of mathematical formulae, concepts, and theorems means that negative attitudes toward maths are common among learners worldwide (OECD, 2013). Despite the many endeavors that have been made to address maths anxiety and avoidance, building positive behaviors and beliefs before negativity emerges may be more productive (Lee and Johnston-Wilder, 2017). Deriving from the field of positive psychology, academic buoyancy and adaptability are two kinds of capacities that can help learners regulate and protect themselves from adverse events or complex situations that arise in a relatively failure-prone learning environment (Collie et al., 2017; Martin and Marsh, 2020). These abilities in maths learning may influence students’ mathematics performance and psychological well-being in later life, thus it is crucial to understand the antecedents of academic buoyancy and adaptability.

Students’ learning skills have been found to be influenced by learning motivation (Hsieh, 2014; Schukajlow et al., 2017). Goal theory (Nicholls, 1984; Ames, 1992), a dominant theoretical perspective on students’ achievement motivations, explains why and how people seek to achieve different goals, including mastery, performance-approach, and performance-avoidance orientations. It provides a framework for studying the contribution of goal orientation to students’ ability to achieve. Although goal orientations have received much attention among educational researchers, few empirical studies have related goal orientations to academic buoyancy and adaptability. To address these research gaps, this study examines the effect of goal orientations on academic buoyancy and adaptability.

Environmental factors can influence both what individuals think and what they do (Schunk and Zimmerman, 2006). Parenting as a crucial home-environment variable may have a salient impact on child development. Darling and Steinberg (1993) developed a contextual model of parenting and posited the linkage between adolescents’ perceptions of parental practices and adolescents’ learning profiles. Based on this model, the present study focused on parents’ involvement in mathematics learning and assumed that students’ perceived parental involvement may influence their goal orientations, academic buoyancy and adaptability.

### Academic buoyancy and adaptability

In educational research (e.g., Li et al., 2017; Sattler and Gershoff, 2019; Wills and Hofmeyr, 2019), much attention has been paid to exploring why some students are successful in school despite experiencing major adversity. This research has produced the well-known concept of academic resilience. A related concept, academic buoyancy, similarly denotes a student’s ability to deal with difficulties and challenges. Compared with resilience, however, buoyancy is more closely related to how students handle daily stressors that a vast majority of students may suffer from, such as urgent deadlines, difficult homework, examination pressure, and unexpectedly or consistently receiving low grades (Martin and Marsh, 2008, 2009). In addition to major adversity (requiring resilience) and everyday challenges and difficulties (requiring buoyancy), new, uncertain, and/or changing circumstances and conditions may arise during learning, leading to the need for another ability, adaptability. According to an early definition by the American Psychological Association, adaptability is “the capacity to make appropriate responses to changed or changing situations; the ability to modify or adjust one’s behavior in meeting different circumstances or different people” (VandenBos, 2007, p. 17). With subsequent research advancements, this concept now includes the ability to make appropriate cognitive, behavioral, and affective adjustments (Martin, 2012; Martin et al., 2012, 2013), or in other words, the ability to adjust one’s thoughts, behaviors, and emotions to navigate new, uncertain, and changing demands.

When examining the development of academic buoyancy and adaptability, it is necessary to consider the cultural values in individual societies. In China, owing to the collectivistic–Confucian cultural tradition, education is perceived as the main means of achieving personal growth and knowledge expansion. Most Chinese parents hope that their children will have a bright future, and are willing to make sacrifices for their children’s education (Lee and Morrish, 2012), and many students agree that academic success can bring honor to the family and repay the parents’ sacrifice and investment (Hau and Salili, 1996; Leung, 2020). Influenced by Confucianism’s belief that diligence, intrinsic motivation, and willpower are more important to personal success than individuals’ innate ability, even though Chinese students have to contend with increasing academic burdens and psychological pressure in competitive academic life, they tend to have high levels of academic buoyancy and adaptability. Considering most of the existing literature base was established in the Western world, the current research aims to generalize the findings of academic buoyancy and adaptability to Confucian culture societies.

### The effect of parental involvement on academic buoyancy and adaptability

Parental involvement in education, also called parental academic involvement, is defined as parents’ engagement with their children’s schools and involvement with their children’s learning to promote their educational success (Hill and Taylor, 2004). It is a multidimensional concept that includes involvement at home, involvement at school, and academic socialization (Fan and Chen, 2001; Hill and Tyson, 2009; Hill et al., 2018). Examples of home-based involvement are assistance with homework and the monitoring of schoolwork and progress. School-based involvement refers to participation in school events and communication with teachers. Academic socialization includes talking to children about schoolwork, their plans, and their goals.

Empirical research has shown that parental involvement can foster student persistence when facing difficulties and challenges (e.g., Wong, 2008; Mena, 2011; Rubach and Bonanati, 2023). For instance, Wong (2008) investigated 171 adolescents in the US to explore the factors that predict students’ ability to adapt and succeed despite adverse circumstances. The results showed that greater parental involvement led to more effort to stay in control and to identify regulations, which helped high-risk adolescents increase their resilience. In addition, many researchers have claimed that engaging parents in an effective mode of academic involvement is an important ingredient for the development of children’s ability to adjust to learning demands arising from unprecedented and changing situations (e.g., Sheldon and Epstein, 2005; Ratelle et al., 2017; Yu et al., 2022). For example, emergency remote instruction for school learning is newly occurring but general during the COVID-19 pandemic. Yu et al. (2022) investigated the contribution of parental involvement during the special period of school closure and found that parental involvement can facilitate children’s learning engagement, which is beneficial for children’s academic performance. Although few studies have directly explored the impact of parental involvement on student academic buoyancy and adaptability, the empirical results above made it appropriate to hypothesize the positive relationship. In the present study, we investigate the association between parental involvement and buoyancy and adaptability in the context of mathematics learning and hypothesize goal orientations may mediate this relationship.

### The mediating roles of goal orientations in the relationship between parental involvement, academic buoyancy, and adaptability

Goal orientations, as part of individual cognitive life, act as frameworks that guide and give purpose to people’s actions (Kaplan and Maehr, 2007). They may reflect a person’s experiences, guide their understanding of events, and produce specific modes of cognition, behavior, and emotion (Elliott and Dweck, 1988). Although researchers hold different opinions on the categorization of goal orientations, “mastery” and “performance” are the two terms that are most commonly used. A mastery goal orientation is defined as a focus on developing competence (Ames, 1992), and it has been found to positively predict outcomes in areas such as task values, self-regulated learning and academic achievement (Fadlelmula et al., 2015; Lazarides et al., 2018; Guo et al., 2022). A performance orientation is defined as a focus on demonstrating competence (Ames, 1992). This has been further divided into “performance-approach” and “performance-avoidance” goal orientations due to different findings about the relationship between performance goal orientations and adaptive outcomes (Elliot, 1997; Elliot and Church, 1997; Elliot, 1999). Specifically, a person with a performance-approach orientation tends to care most about demonstrating a high level of competence and being successful, whereas someone with a performance-avoidance orientation tends to focus on how to avoid showing low competence and encountering failure.

Mastery and performance goal orientations are viewed as developing within a person’s proximal environment and thus influenced by factors in that environment (Régner et al., 2009; Zheng et al., 2019). Several empirical studies have shown that parental involvement has various consequences for students’ different types of goal orientations. For mastery goal orientations, many studies have drawn consistent results about the positive impact of parental involvement (e.g., Duchesne and Ratelle, 2010; Wang et al., 2019; Li et al., 2020). For example, Li et al. (2020) investigated 3,378 Chinese adolescents and found that when they perceived more involvement and autonomy support by parents, they have the higher levels of mastery goals. However, findings about the effect of parental involvement on performance goal orientations are mixed. Luo et al. (2013) used a sample of 1,667 Singaporean students to investigate the role of parenting behaviors in students’ development of goal orientations and found that parental involvement in learning modestly and positively predicted students’ performance-approach and performance-avoidance goal orientations. Zong et al. (2018) found that parental involvement significantly and positively predicted students’ performance-approach goal orientations but had non-significant effects on performance-avoidance goal orientations. Other studies have found a negative correlation between parental involvement and students’ performance-avoidance goal orientations (e.g., He et al., 2015). These inconsistent findings suggest that more studies are needed to examine the relationship between parental academic involvement and students’ goal orientations.

The effects of different kinds of goal orientations on students’ academic progress and outcomes have been examined. Many studies have confirmed the positive effect of mastery goals on student learning. For example, Skaalvik (2018) found that a mastery goal orientation was a strong and direct predictor of the use of problem-focused coping strategies. Additionally, this orientation predicted lower levels of math anxiety and reduced use of self-protective coping strategies, which are conceptualized as maladaptive. Yu and Martin (2014) recruited a sample of 3,753 school children in China and found that mastery goals were positively correlated with students’ motivation, engagement, and academic buoyancy. Findings about the relationship between performance goals and adaptive outcomes are inconsistent. Most research has suggested that performance-approach goals can facilitate learning but that performance-avoidance goals inhibit learning (Luo et al., 2013; Mouratidis et al., 2018; Ng, 2018; Möcklinghoff et al., 2023). However, some research has reported that both performance-approach and performance-avoidance goals positively predict academic achievement, engagement, and deep learning strategies (King, 2016), while other researchers have argued that both types of performance orientation underlie a maladaptive response pattern (Dweck, 1986; Lee et al., 2021). Although these inconsistent results mean that the effects of performance goals on student learning are still an open question, they show that goal orientations can predict some coping effects related to academic buoyancy and adaptability. Therefore, it is possible to speculate about the influence of goal orientations on students’ academic buoyancy and adaptability. Given the possible relationship between parental involvement and goal orientations and the influence of goal orientations on academic buoyancy and adaptability, we expect the three types of goal orientation partially mediate the hypothesized relationship between parental involvement and academic buoyancy and adaptability.

### Influence of control variables

In examining the relationship between parental involvement, goal orientations, academic buoyancy, and adaptability, it is essential to control potentially confounding factors. Researchers have found that family socioeconomic status (SES) has some effects on the degree of parental involvement and that students with a higher family SES perceive greater parental involvement (e.g., Hoff et al., 2002). Moreover, family SES has an effect on students’ development of goal orientations (Xu et al., 2020); parental educational level in particular is positively associated with students’ mastery and performance-approach goal orientations (Mouratidis et al., 2018). Furthermore, research has shown that a higher family SES seems to be a protective factor when a student is exposed to learning difficulties, so high SES students tend to have greater academic buoyancy and adaptability (Yu et al., 2019). Thus, family SES – obtained by integrating parental education, parental occupation, and family learning resources to give a composite score – is included as a covariate in this study.

Meanwhile, the four variables in the current research have been found to vary depending on gender. Some researchers found that parents were involved in their sons’ and daughters’ academic learning in significantly different ways, and that girls reported more parental involvement (e.g., Muller, 1998; Shapira-Lishchinsky and Zavelevsky, 2020). Besides, certain gender differences emerged in goal orientations. Boys were more likely to adopt a performance goal orientation (approach or avoidance), while girls were more likely to adopt a mastery goal orientation (D’Lima et al., 2014). Moreover, gender has a significant relationship with academic buoyancy: boys tend to have significantly higher scores for buoyancy than girls (Datu and Yang, 2018). Gender has also been found to have specific effects on affective adaptability, which is a sub-constructs of adaptability, with boys demonstrating greater constructive affective regulation (Martin et al., 2012). Therefore, gender is taken as another covariate in the present study.

### The current study

**Figure 1:**
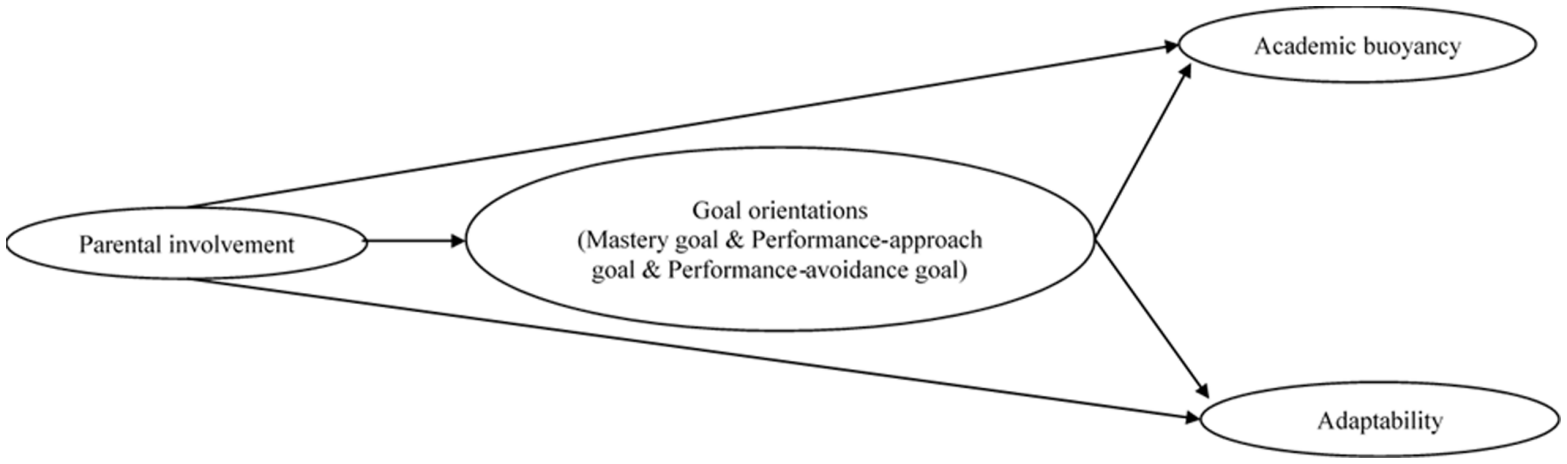
Proposed theoretical model of parental involvement, goal orientations, academic buoyancy and adaptability.

The current research aims to combine the contextual model of parenting with goal theory to test the effects of parental involvement on students’ goal orientations, academic buoyancy, and adaptability in the specific context of maths learning in China. Figure 1 depicts a conceptual model summarizing the proposed relationships. Specifically, we propose and test the following hypotheses: (1) parental involvement is a positive predictor of academic buoyancy and adaptability; (2) parental involvement is a positive predictor of mastery goal orientation and performance-approach goal orientation, and a negative predictor of performance-avoidance goal orientation; (3) goal orientations mediate the association between parental involvement and students’ academic buoyancy and adaptability.

The study contributes to both theory and practice in the following ways. First, it investigates students’ reactions to academic challenges and changes by considering the role of Chinese cultural values and social context. Furthermore, it enriches research by examining the mediational mechanism of students’ purposes and behaviors to seek to achieve different goals underlying the links between parental involvement in education and students’ coping abilities from a domain-specific perspective. This is still a research gap in the literature. Finally, the research can inform efforts to develop adolescents’ academic buoyancy and adaptability and guide policymakers, teachers, and parents in encouraging a deep desire for learning when students are faced with challenges and changes when learning mathematics.

## Methods

### Participants and procedure

The sample comprises 1,164 students along with their parents/guardians from seven secondary schools in a first-tier city in China. Among the students, 589 (50.6%) were boys and 575 (49.4%) were girls. The selection of schools was based on school principals’ willingness to collaborate with the research following their receipt of an invitation letter. Three were boarding schools and four were day schools. In each school, between four and eight classes of eighth-grade classes were randomly selected to complete a survey. All the students and their parents/guardians completed and submitted consent forms before the survey was administered. Once the student and parent/guardian consent had been obtained, the students were asked to fill out a 20-min questionnaire in class, and their parents/guardians were asked to complete a 10-min questionnaire about family SES at home.

### Measures

#### Parental involvement

The Parents’ Involvement in Children’s Learning Scale (Cheung and Pomerantz, 2011) was used to measure the students’ perceived parental involvement. The original scale consisted of 10 items. We modified some items to focus on the subject of mathematics. An example item was “My parents try to get to know the maths teachers at my school”. The participants rated the items on a 7-point Likert scale from 1 (strongly disagree) to 7 (strongly agree). A higher score represented greater parental involvement. In the subsequent analysis, according to the cut-off value suggested by Stevens (1992), the factor loading of item 6 was found to be lower than 0.4, so item 6 was excluded. Cronbach’s alpha for the modified nine-item scale was 0.877.

#### Goal orientation

We measured the students’ goal orientations based on the Goal Orientation Scale (Midgley et al., 2000). This scale consisted of 14 items. Some were adjusted to concentrate on mathematics learning. Five items focused on mastery goal orientation (e.g., “It’s important to me that I learn a lot of new maths concepts this year”). Five items focused on performance-approach goal orientation (e.g., “One of my goals is to show others that I’m good at maths”). Four items focused on performance-avoidance goal orientation (e.g., “It’s important to me that I do not look stupid in maths class”). The items were rated using a 7-point Likert scale ranging from 1 (strongly disagree) to 7 (strongly agree). A higher score represented a higher level of goal orientation. The values of Cronbach’s alpha for the subscales measuring mastery goal orientation, performance-approach goal orientation, and performance-avoidance goal orientation were 0.915, 0.913, and 0.867, respectively.

#### Academic buoyancy

The students’ academic buoyancy in mathematics learning was measured using the four-item Academic Buoyancy Scale developed by Martin and Marsh (2008). We adjusted the items to concentrate on mathematics learning (e.g., “I do not let the stress of studying maths get on top of me”). The participants rated items using a 7-point Likert scale ranging from 1 (strongly Disagree) to 7 (strongly Agree). A higher score demonstrates greater academic buoyancy. The Cronbach’s alpha for this scale was 0.868.

#### Adaptability

The adaptability scale was adapted from the previously developed Adaptability Scale (Martin et al., 2012, 2013). Six statements focused on cognitive–behavioral adaptability (e.g., “While learning maths, I am able to adjust my thinking or expectations to assist me in a new situation if necessary”), while the other three statements focused on affective adaptability (e.g., “While learning maths, I am able to reduce negative emotions to help me deal with uncertain situations’’). The students responded to the items using a 7-point Likert scale ranging from 1 (strongly disagree) to 7 (strongly agree). Higher scores for this measure indicated greater adaptability. Cronbach’s alpha for this scale was 0.931.

#### Student gender and family SES

Student gender (0 = female; 1 = male) and family SES were included in the investigation. Family SES was reported by the parents or guardians, and included parental education, parental occupation, and family learning resources. Parents’ education level was measured by collecting data on the highest level of education completed (e.g., 0 = no education, 1 = elementary school, 2 = junior high school, and 3 = high school). Parents’ occupation was determined according to information about the occupation and job category provided by the participants. Based on the Chinese Occupational Prestige Measuring Index (COPMI; Li, 2005), we categorized occupations and jobs into seven levels, and individuals at the same level were given identical scores (with scores ranging from 7 to 1). The highest level included occupations such as engineer and university teacher, and the lowest level included jobs such as porter and nanny. The maximum of the mother’s and the father’s educational (or occupational) score was viewed as the score of the parents’ education (or occupation). Besides, the participants needed to report the family resources for student learning, such as the availability of a desk at home to study at, the student’s own room, and online maths course resources. We first obtained a summary index of family learning resources by calculating standardized z-scores; we then subjected the parental education, parental occupation, and family learning resources to factor analysis using the principal component method of extraction to determine an aggregate score for family SES. A higher score demonstrated a higher family SES.

### Analyses overview

In the hypothesized model, the independent variable was parental involvement. The mediating variables were the three types of goal orientation. The dependent variables were academic buoyancy and adaptability. Student gender and family SES were the two control variables. Descriptive statistics and correlations of all the variables were obtained using SPSS 21.0 software.

A two-step method was used to test the hypothesized model in Mplus 7.4. First, we conducted confirmatory factor analysis (CFA) to confirm the factorial structures of the scales measuring parents’ involvement in children’s learning, goal orientations, academic buoyancy, and adaptability (Wang and Wang, 2012). The measurement model was a six-factor model consisting of parental involvement, mastery goals, performance-approach goals, performance-avoidance goals, academic buoyancy, and adaptability. All six factors were allowed to relate to each other. Second, structural equation modeling (SEM) was used to assess the mediation. We tested the indirect effects by conducting bootstrap analyses with 95% confidence intervals (bootstrap replications: 1,000) (Preacher and Hayes, 2008). Confidence intervals not containing 0 indicated significant mediated effects. The full information maximum likelihood method was used to handle missing values (Schlomer et al., 2010). The model was considered an adequate fit based on the following indices: the root mean square error of approximation (RMSEA) and standardized root mean square residual (SRMR) were all less than 0.08 and the Tucker-Lewis index (TLI) and comparative fit index (CFI) were all greater than 0.90 (Schermelleh-Engel et al., 2003). All the loading values of the items on the latent factors were required to be higher than 0.4.

## Results

### Descriptive statistics and correlations

Table 1 shows the descriptive statistics and correlations between the study variables. Both academic buoyancy and adaptability were positively related to parental involvement (*r* = 0.301 and *r* = 0.399, *p* < 0.001, respectively), mastery goals (*r* = 0.447 and *r* = 0.557, *p* < 0.001, respectively), and performance-approach goals (*r* = 0.143 and *r* = 0.192, *p* < 0.001, respectively). Parental involvement was positively associated with mastery goals (*r* = 0.355, *p* < 0.001) and performance-approach goals (*r* = 0.119, *p* < 0.001). Gender was positively correlated with performance-approach goals (*r* = 0.125, *p* < 0.001), performance-avoidance goals (*r* = 0.093, *p* < 0.01), academic buoyancy (*r* = 0.217, *p* < 0.001), and adaptability (*r* = 0.207, *p* < 0.001). Family SES was positively associated with parental involvement (*r* = 0.250, *p* < 0.001), mastery goals (*r* = 0.071, *p* < 0.05), academic buoyancy (*r* = 0.100, *p* < 0.001), and adaptability (*r* = 0.100, *p* < 0.001), but negatively associated with performance-approach goals (*r* = −0.089, *p* < 0.01) and performance-avoidance goals (*r* = −0.128, *p* < 0.001).

**Table 1:** Descriptive statistics and correlation results of study variables (*N* = 1,164).

	1	2	3	4	5	6	7	8
1 Gender	–							
2 Family SES	−0.023	–						
3 Parental involvement	−0.027	0.250***	–					
4 Mastery goal	−0.004	0.071*	0.355***	–				
5 Performance-approach goal	0.125***	−0.089**	0.119***	0.139***	–			
6 Performance-avoidance goal	0.093**	−0.128***	−0.044	−0.022	0.628***	–		
7 Academic buoyancy	0.217***	0.100***	0.301***	0.447***	0.143***	−0.017	–	
8 Adaptability	0.207***	0.100***	0.399***	0.557***	0.192***	0.004	0.793***	–
*M*	0.51	0.000	5.061	5.994	3.561	3.452	5.111	5.182
*SD*	0.500	1.000	1.186	1.005	1.416	1.399	1.348	1.157

*p < 0.05, **p < 0.01, ***p < 0.001.

### Testing the measurement model

When the measurement model was tested using six factors related to their observed indicators, the CFA results showed that *χ*^2^ (576) = 2994.064, CFI = 0.916, TLI = 0.908, RMSEA = 0.060 and SRMR = 0.055, indicating a good model fit for the four scales. Moreover, the standardized factor loadings of each indicator on the corresponding latent construct were all above 0.4 and significant at the *p* < 0.001 level, suggesting that the observed indicators were strongly loaded on their respective latent variables.

### Examining the structural model

**Figure 2:**
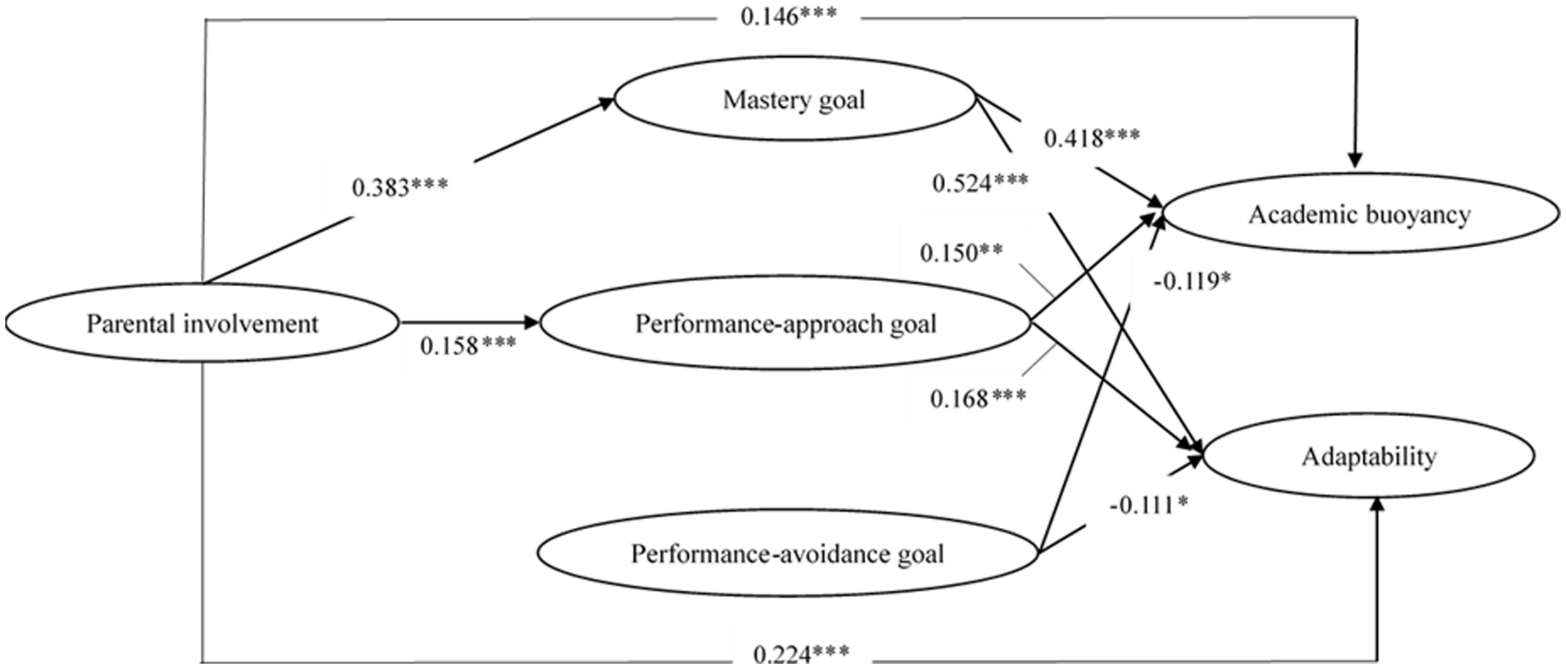
Structural equation model testing the relationship between parental involvement, goal orientations, academic buoyancy, and adaptability. To simplify the view, we do not show the observed indicators of each latent variable in the figure. All the correlations and path coefficients shown in the figure are standardized and statistically significant (*p < 0.05, **p < 0.01, ***p < 0.001).

The SEM results are presented in Figure 2 and demonstrate an acceptable model fit: *χ*^2^ (711) = 3318.021, CFI = 0.912, TLI = 0.903, RMSEA = 0.056, SRMR = 0.053. The total R squares for academic buoyancy and adaptability were 0.356 and 0.529, respectively. Table 2 shows the standardized estimates and 95% confidence intervals of both direct and indirect paths. The direct path between parental involvement and academic buoyancy was significant (*β* = 0.146, *p* < 0.001). The mediation effect of a mastery goal orientation between parental involvement and academic buoyancy was 0.160 (*p* < 0.001), indicating that a mastery goal orientation had a partial positive mediating effect on the correlation between parental involvement and academic buoyancy. The mediation effect of a performance-approach goal orientation between parental involvement and academic buoyancy was 0.024 (*p* < 0.05), indicating that the performance-approach goal orientation had a partial positive mediating effect on the correlation between parental involvement and academic buoyancy. The mediation effect of the performance-avoidance goal between parental involvement and academic buoyancy was not significant (*β* = 0.000, *p* > 0.05), indicating that the performance-avoidance goal did not mediate between parental involvement and academic buoyancy. Parental involvement explained 33% of the variance of academic buoyancy. Indirect links accounted for 55.8% of the relationship between parental involvement and academic buoyancy, with a stronger link through mastery goals (48.5%) than through performance-approach goals (7.3%).

**Table 2:** The results of the bootstrap analysis (*N* = 1,164).

Paths	β	95% CI	
		Low	High
Parental Involvement – Academic Buoyancy	0.146***	0.059	0.222
Parental Involvement – Mastery Goal – Academic Buoyancy	0.160***	0.113	0.207
Parental Involvement – Performance-Approach Goal – Academic Buoyancy	0.024*	0.007	0.047
Parental Involvement – Performance-Avoidance Goal – Academic Buoyancy	0.000	−0.010	0.011
Total effect	0.330***	0.256	0.402
Parental Involvement – Adaptability	0.224***	0.139	0.298
Parental Involvement – Mastery Goal – Adaptability	0.201***	0.149	0.257
Parental Involvement – Performance-Approach Goal – Adaptability	0.027**	0.011	0.050
Parental Involvement – Performance-Avoidance Goal – Adaptability	0.000	−0.010	0.011
Total effect	0.451***	0.373	0.521

*p < 0.05, **p < 0.01, ***p < 0.001.

The direct path between parental involvement and adaptability was significant (*β* = 0.224, *p* < 0.001). The mediation effect of a mastery goal orientation on the relationship between parental involvement and adaptability was 0.201 (*p* < 0.001), indicating that a mastery goal orientation had a partial positive mediating effect on the correlation between parental involvement and adaptability. The mediation effect of the performance-approach goal orientation between parental involvement and adaptability was 0.027 (*p* < 0.01), indicating that a performance-approach goal orientation had a partial positive mediating effect on the correlation between parental involvement and adaptability. The mediation effect of a performance-avoidance goal orientation between parental involvement and adaptability was not significant (*β* = 0.000, *p* > 0.05), indicating that this orientation did not mediate between parental involvement and adaptability. Parental involvement explained 45.1% of the variance of adaptability. Indirect links accounted for 50.6% of the relationship between parental involvement and adaptability, with a stronger link through mastery goals (44.6%) than through performance-approach goals (6.0%).

## Discussion

This study applied the integration of the contextual model of parenting and goal theory as the theoretical foundation to examine the relationship between parental involvement, goal orientations, academic buoyancy, and adaptability in maths learning. The results indicated that the students’ perceived parental involvement was positively related to their academic buoyancy and adaptability. Furthermore, parental involvement was significantly associated with the students’ self-reported mastery and performance-approach goal orientations, which further mediated the relationship between parental involvement and academic buoyancy, as well as the relationship between parental involvement and adaptability. However, the mediating role of a performance-avoidance goal orientation in the relationship was not significant. Our findings provide evidence of the parental and motivational antecedents of academic buoyancy and adaptability in Chinese society and maths learning. The findings and implications are discussed below.

### Parental involvement and academic buoyancy and adaptability

The extent of parental involvement perceived by the students positively predicted both academic buoyancy and adaptability. This association indicates that when students perceive greater parental involvement, they are likely to be more competent in dealing with learning difficulties, challenges, uncertainty, and novelty in maths classrooms. These results are consistent with the literature on the influence of parents’ behavior on students’ mathematical learning abilities (Levine et al., 2010; Ramani et al., 2015), and especially, are in line with the previous findings about the parents’ involvement in benefiting students’ perceived competence (Fan and Williams, 2010; Núñez et al., 2019). A possible explanation for the contribution of parents’ learning-related practices to their children’s academic functioning could be found from the perspective of the beliefs held by both parents and children in the Chinese context, shaped by Confucianism and social norms. For Chinese parents, most of them believe that self-improvement is a primary goal of education and they should be responsible for their children’s development. Especially, given the critical role of mathematics in preparing youngsters for future employment and personal development (Tan, 2013), they highlight mathematics achievement as being of great importance and are motivated to engage more with their children’s maths learning. Influenced by some well-known Chinese sayings, such as “The sharpness of a sword comes from being tempered, and the fragrance of plum blossoms comes from the bitter cold”, they consider their children’s adaptive responses to complex learning situations as signs that diligence is developing. When children come across difficulties or any situations never encountered before in maths learning, parents are willing to provide behavior and emotional support, which benefits the development of children’s academic buoyancy and adaptability. For Chinese students, the ideas that academic success can bring honor to the whole family and is a demonstration of filial piety (Ng and Wang, 2019) and their perceptions of the value their parents place on effort and persistence, may strengthen the process of internalization needed to cope with challenges, difficulties, novelty, and uncertainty in the maths learning process. This then means that they are more competent academically and prioritize both effort and academic outcomes.

### Parental involvement and goal orientations

This research identified positive correlations between parental involvement and two goal orientations (mastery and performance-approach goal orientations), in line with the literature (e.g., Gonzalez and Wolters, 2006; Duchesne and Ratelle, 2010; Zong et al., 2018). As discussed above, high expectations of academic achievement and parents’ responsibility for training their children are promoted in Chinese society, based on Confucianism. The beneficial effect of parental practices on students’ motivation to achieve may be the result of students seeing their parents taking a great interest in their education and getting actively involved in their maths learning. The children get the message that their parents are committed to the young person’s academic ability and success, and may conclude that growth and self-improvement are their primary responsibility. This autonomy fosters the development of students’ mastery orientation. Meanwhile, greater parental involvement may elicit students’ mindset that they need to excel academically and outperform peers, strengthening their performance-approach goal orientation (Gonzalez and Wolters, 2006).

Contrary to our hypothesis, there was no significant correlation observed between parental involvement and performance-avoidance goal orientation, but it is consistent with the findings of Zong et al. (2018). This finding suggests that the effect of the avoidance-oriented motivation provided by parental involvement may dissipate when learning maths. This pattern may be due to China’s newly released compulsory education policy. This is called ‘‘the double reduction policy’’ and it aims to ease the learning burden and examination pressure on students by adjusting homework assignments, reducing the importance of test scores, and prohibiting after-school tutoring. Improving maths skills to support students’ future learning and career development is a major pillar of the current reform of mathematics education in China. As a result, regardless of the extent of parental involvement, excessive concerns about demonstrating poor academic competence and failure in education have generally declined among Chinese junior high school students. Their avoidance tendency may have become independent of parental intervention.

### The mediating roles of goal orientations

The path analysis showed that the mastery goal and performance-approach goal orientations significantly and partially mediated the relationship between parental involvement and academic buoyancy, as well as adaptability (i.e., greater parental involvement is indirectly linked to higher academic buoyancy and adaptability by fostering both mastery and performance-approach goal orientations). The mediating role of the two goal orientations found in this study is partially consistent with the contextual model of parenting and goal theory discussed above. Parental behaviors can influence learners’ learning motivation (Darling and Steinberg, 1993). When parents get involved in their children’s maths learning, they may provide encouragement and praise for good mathematics performance, show that they have high expectations, and highlight the importance of self-improvement. These behaviors may create a cognitive and psychological environment in which students understand that they need to develop and prove their ability and realize the importance of academic success. Then, these goal orientations may be beneficial for students to trigger adaptive coping motivation when dealing with complex problems in learning (Kaplan and Maehr, 2007). To be specific, students who endorse the goals of developing and demonstrating competence and pursuing academic success may also show task persistence, preference for challenges, and ease with uncertainty. However, the performance-avoidance goal orientation did not mediate the relationship, which is inconsistent with the findings of previous studies regarding its mediating effect on the relations between parental behaviors and students’ academic functioning (e.g., Chen, 2015; Xiang et al., 2017; Xu et al., 2018). This may be because parental involvement in mathematics learning helps students develop their perceived control over their abilities. In challenging or changing situations, students who benefit from sufficient parental involvement in their education may believe that only adaptive and positive adjustments in response to those situations will assist them in attaining academic success. A performance-avoidance goal orientation does not need to be psychologically prominent for these adjustments to occur. The function of performance-avoidance goals should be explored further in the future.

## Limitations and future research directions

Despite its significant findings, the study has a few limitations that should be acknowledged. First, the information about the relevant variables was self-reported by the participants in response to a questionnaire. Although it is assumed that self-reported data reflect participants’ actual thoughts and behavior, participants’ responses may sometimes be inconsistent with their actions. Future studies should adopt various assessments, such as observations or interviews to triangulate the data. Second, this research measured parental involvement by adopting a unidimensional scale. Considering multiple aspects of parental involvement may have different effects on students’ mathematics learning, future research can use the multidimensional measurement of parental involvement and avoid broadly defining this concept. Third, this study used a cross-sectional design to test the research hypotheses. Although the method is efficient and accurate, it is impossible to explore the correlations over time and understand the causality between the variables. Future work should use longitudinal investigations to test the causal associations. Fourth, only 8th-grade students and their parents/guardians were recruited for this research; care should be taken in extending the results to individuals in different school years. As for directions for future work, studies should recruit more representative and diverse samples.

## Implications and conclusions

Our findings suggest that the degree of parents’ involvement in mathematics learning and students’ beliefs about the reasons for achieving success can act as protective factors for students when faced with academic difficulties, challenges, and new situations of any kind. It is important to encourage parents to spend more time providing effective support for their children’s maths learning activities. Schools should increase opportunities to boost parental involvement by organizing training activities and communications with parents about students’ progress. Teachers could launch public workshops to offer tips to help with mastering maths, coping strategies for learning challenges, and, most importantly, what parents can do to cultivate their children’s maths learning abilities. In addition, educators could seek to motivate students to trigger their psychological autonomy and develop healthy goal orientations.

In conclusion, this is the first study to examine both the family and individual antecedents of academic buoyancy and adaptability in the maths learning field. It supports our conjecture about the unique roles of mastery and performance-approach goal orientations as mediators of the relationship between parental involvement and academic buoyancy and adaptability. The findings hint at the potential roles played by culture and social context and also contribute to a more complete portrait of parental impact on students’ proficiency in learning and the motivational process behind it. We hope that our findings will provide a useful foundation for future studies of the development of students’ academic buoyancy and adaptability.

## Data availability statement

The original contributions presented in the study are included in the article/supplementary material, further inquiries can be directed to the corresponding author.

## Ethics statement

The studies involving human participants were reviewed and approved by the University of Hong Kong’s Human Research Ethics Committee (HREC). Written informed consent to participate in this study was provided by the participants’ legal guardian/next of kin.

## Author contributions

MC conducted the survey, analyzed the data, and wrote the manuscript. IM supervised the investigating, analyzing, and writing process. All authors contributed to the article and approved the submitted version.

## Conflict of interest

The authors declare that the research was conducted in the absence of any commercial or financial relationships that could be construed as a potential conflict of interest.

## Publisher’s note

All claims expressed in this article are solely those of the authors and do not necessarily represent those of their affiliated organizations, or those of the publisher, the editors and the reviewers. Any product that may be evaluated in this article, or claim that may be made by its manufacturer, is not guaranteed or endorsed by the publisher.
